# Metronomic Chemotherapy for Advanced Prostate Cancer: A Literature Review

**DOI:** 10.3390/jcm11102783

**Published:** 2022-05-15

**Authors:** Shruti Parshad, Amanjot K. Sidhu, Nabeeha Khan, Andrew Naoum, Urban Emmenegger

**Affiliations:** 1 Division of Medical Oncology, Odette Cancer Centre, Sunnybrook Health Sciences Centre, Toronto, ON M4N 3M5, Canada; shruti.parshad@uwaterloo.ca (S.P.); ak7sidhu@edu.uwaterloo.ca (A.K.S.); nabeeha.khan@sri.utoronto.ca (N.K.); andrew.naoum@sri.utoronto.ca (A.N.); 2 Biological Sciences Research Platform, Sunnybrook Research Institute, Sunnybrook Health Sciences Centre, Toronto, ON M4N 3M5, Canada; 3 Department of Medicine, University of Toronto, Toronto, ON M5S 1A1, Canada; 4 Institute of Medical Science, University of Toronto, Toronto, ON M5S 1A1, Canada

**Keywords:** metronomic chemotherapy, metastatic castration-resistant prostate cancer, cyclophosphamide, side effects

## Abstract

Metastatic castration-resistant prostate cancer (mCRPC) is the ultimately lethal form of prostate cancer. Docetaxel chemotherapy was the first life-prolonging treatment for mCRPC; however, the standard maximally tolerated dose (MTD) docetaxel regimen is often not considered for patients with mCRPC who are older and/or frail due to its toxicity. Low-dose metronomic chemotherapy (LDMC) is the frequent administration of typically oral and off-patent chemotherapeutics at low doses, which is associated with a superior safety profile and higher tolerability than MTD chemotherapy. We conducted a systematic literature review using the PUBMED, EMBASE, and MEDLINE electronic databases to identify clinical studies that examined the impact of LDMC on patients with advanced prostate cancer. The search identified 30 reports that retrospectively or prospectively investigated LDMC, 29 of which focused on mCRPC. Cyclophosphamide was the most commonly used agent integrated into 27/30 (90%) of LDMC regimens. LDMC resulted in a clinical benefit rate of 56.8 ± 24.5% across all studies. Overall, there were only a few non-hematological grade 3 or 4 adverse events reported. As such, LDMC is a well-tolerated treatment option for patients with mCRPC, including those who are older and frail. Furthermore, LDMC is considered more affordable than conventional mCRPC therapies. However, prospective phase III trials are needed to further characterize the efficacy and safety of LDMC in mCRPC before its use in practice.

## 1. Introduction

Cancer is amongst the most significant contributors to disease burden worldwide, with cancer incidence expected to double by 2035 [1]. The global cancer burden is greatest in low- and middle-income countries (LMIC), in which cancer incidence is rising most rapidly, where 75% of cancer deaths occur, yet where only 5% of the global spending on cancer is directed [1]. Prostate cancer follows this general trend as the second most common malignancy in men globally [2].

Localized prostate cancer is highly curable, but metastatic prostate cancer remains a fatal condition to date [3]. While prostate cancer is diagnosed at a median age of 66, prostate cancer-related deaths occur at a median age of 80 [4].

Because prostate cancer is an androgen-driven disease, androgen deprivation therapy (ADT) is the usual first-line therapy for metastatic prostate cancer, nowadays often combined with either docetaxel chemotherapy or androgen receptor signaling inhibitors (ARSi) such as abiraterone, apalutamide, or enzalutamide [5]. However, such patients will develop ADT-resistant prostate cancer eventually, referred to as metastatic castration-resistant prostate cancer (mCRPC), the lethal form of prostate cancer.

Docetaxel was established as the first life-prolonging and quality-of-life-improving therapy for mCRPC in 2004, providing a survival benefit of around three months [6,7]. This intravenous chemotherapeutic of the taxane family is usually administered as a three-weekly regimen at the maximum tolerated dose (MTD), which is associated with numerous acute and chronic side effects (e.g., myelosuppression, mucositis, and peripheral neuropathy). Thus, docetaxel is often not considered for older men who may have a lower tolerance than younger, healthier patients [8,9,10,11].

In recent years, abiraterone and enzalutamide have been approved as mCRPC treatment options, typically used in first line [3]. Both agents are relatively well tolerated, but acquired therapeutic resistance is the ultimate outcome of ARSi therapy in most instances [12,13]. Radium-223 and cabazitaxel chemotherapy represent treatment options for ARSi- and docetaxel-resistant mCRPC [14,15]. However, access to all these life-prolonging yet expensive prostate cancer therapies is limited, notably in LMIC [16,17]. Hence, there is a continued need for affordable mCRPC therapies, especially those suitable for the typically elderly and often patients with advanced prostate cancer who are frail. Low-dose metronomic chemotherapy (LDMC) possess many characteristics to fill this unmet need. 

LDMC refers to the continuous administration of low doses of conventional chemotherapy drugs over a long period, usually via daily oral intake without scheduled treatment breaks, resulting in antiangiogenic and immunomodulatory anti-tumor effects amongst others [18]. Due to the low drug doses used, LDMC has a superior toxicity profile compared with MTD chemotherapy, including in people who are older and frail [19,20]. Furthermore, LDMC is relatively affordable owing to the use of off-patent drugs such as cyclophosphamide (CPA) and modest needs for monitoring treatment-associated side effects [21,22]. Herein, we summarize the findings of 30 studies on the role of LDMC in advanced prostate cancer. 

## 2. Materials and Methods 

### 2.1. Search Strategy and Study Selection

**Figure 1:**
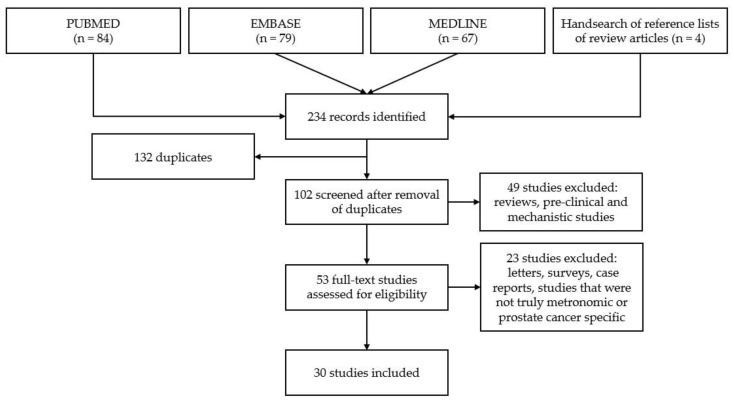
Flow diagram of search strategy.

A systematic search of the PubMed, EMBASE, and MEDLINE electronic databases was conducted from inception up to 31 December 2021 to identify all relevant studies investigating the clinical impact of LDMC in patients with prostate cancer. The search strategy involved combining a methodological filter to specifically identify ‘full text articles’ using the search terms ‘metronomic’ and ‘prostate cancer.’ English-written literature was highly valued in the conduct of this review due to the ease of data extraction. However, non-English language was not a reason for exclusion. Additional studies were identified by screening the reference lists of review articles on LDMC. Titles and abstracts were screened for eligibility. The exclusion criteria are highlighted in Figure 1. Treatment regimens comprising at least one component administered without prespecified breaks were considered metronomic. 

### 2.2. Data Extraction and Statistical Analysis 

Two independent reviewers extracted information on study type and design, country of study, number of patients, patient demographics, treatment details, response criteria used and response rates, survival data, adverse events, and fatalities. Statistical analyses were computed using RStudio (RStudio for Macintosh, version 1.1.463). Graphs were created with Microsoft^®^ Excel for Mac 16.45 (www.microsoft.com), Draw.io (Version 16.5.1; https://app.diagrams.net), or GraphPad Prism (Version 9.3.1; https://www.graphpad.com). 

## 3. Results

### 3.1. Study Selection 

Among the 234 reports identified during the initial search, there were 132 duplicates, leaving 102 abstracts for screening. Following the removal of 49 studies comprising reviews, and pre-clinical and mechanistic studies, we analyzed the full text of 53 articles. A further 23 studies were excluded, including letters, surveys, case reports, and studies that were not truly metronomic or prostate cancer specific. Overall, we identified 30 studies on the clinical use of LDMC for prostate cancer, as depicted in Figure 1. Key study details are outlined in Table 1. 

### 3.2. Study and Patient Characteristics

**Figure 2:**
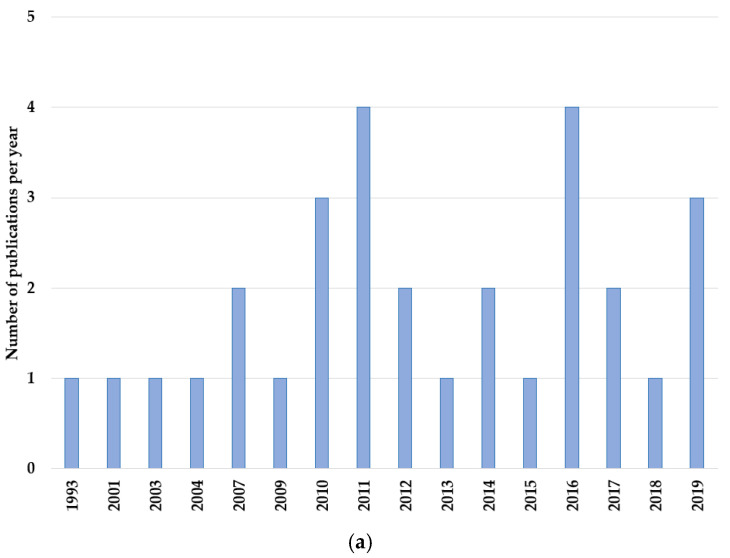

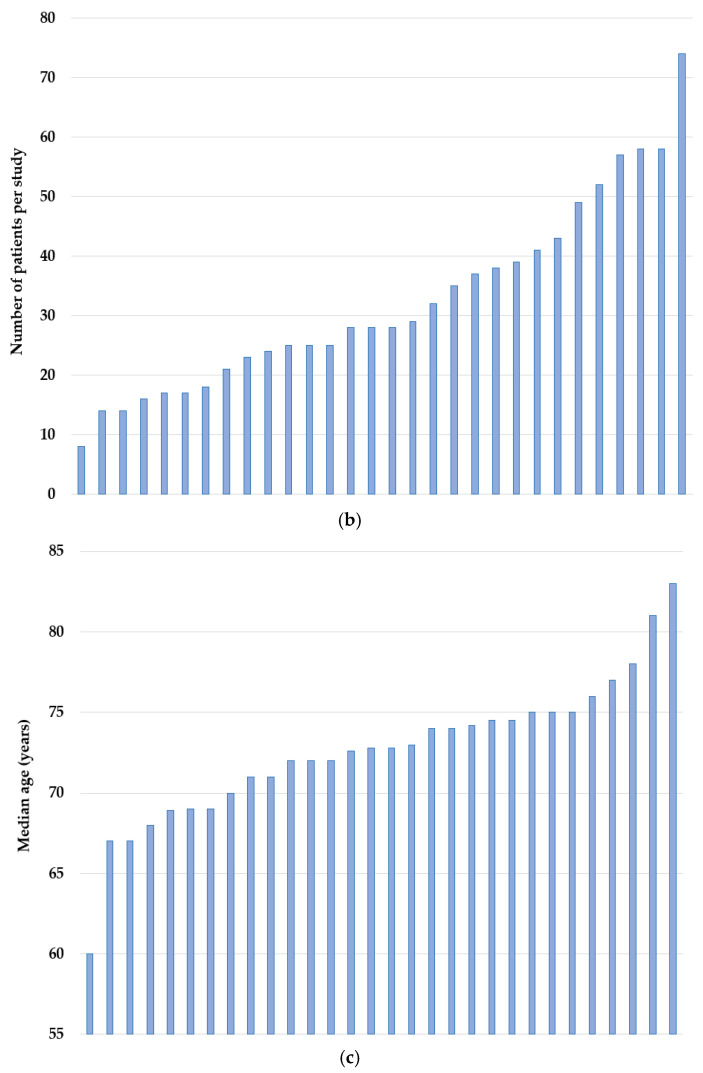
(**a**) Number of LDMC publications per year. (**b**) Number of patients per study. (**c**) Median patient age in years per study.

LDMC studies in the field of prostate cancer cover the time period from 1993 until 2019, with the majority (23/30; 77%) published from 2010 onwards (Figure 2a). More than half of the studies analyzed were conducted in Europe (17/30; 57%), notably with ten of those (33% of all studies) in Italy. Moreover, eight studies (27%) were conducted in Asia, whereas only five reports (17%) were from North America and none were from Asia or South America. Eleven studies (37%) were retrospective analyses. Among the nineteen prospective studies, two (11%) were phase I [27,36], one (5%) was phase I/II [47], and sixteen (84%) were phase II studies [22,25,26,29,30,33,34,37,38,40,41,42,43,45,48,49]. Across both prospective and retrospective phase II type studies, the majority (23/26, 88%) were single arm trials [19,21,22,23,25,26,28,29,30,31,32,33,35,37,38,40,41,42,44,48,49]. 

**Figure 3:**
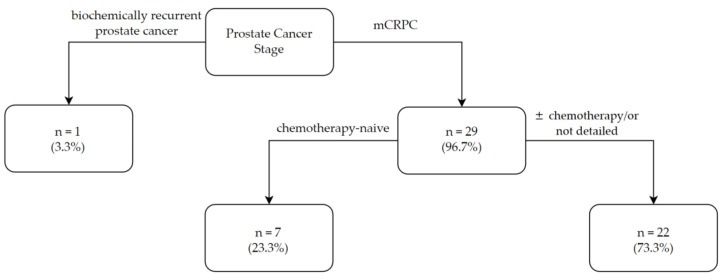
Prostate cancer stage of patients of LDMC studies. mCRPC: metastatic castration-resistant prostate cancer.

The 30 studies comprise information on 973 patients overall, with 28 (range 8 to 74) being the median number of patients per study (Figure 2b). The median patient age per trial ranged from 60 to 83 years, of which 72.8 years was the median of the reported medians (Figure 2c). Of the 30 studies included, only one reported on men with biochemically recurrent (i.e., non-metastatic and castration-sensitive) prostate cancer (Figure 3) [22]. All other studies focused on patients with mCRPC and variable treatment history. Seven (23%) studies included chemotherapy-naïve participants [25,32,33,38,45,46,48], three reported on study subjects with or without prior chemotherapy (typically docetaxel) exposure [28,31,42], and nineteen did not provide details on prior therapies other than the use of ADT [19,21,23,24,26,27,29,30,34,35,36,37,39,40,41,43,44,47,49]. 

### 3.3. Metronomic Treatment Regimens

**Figure 4:**
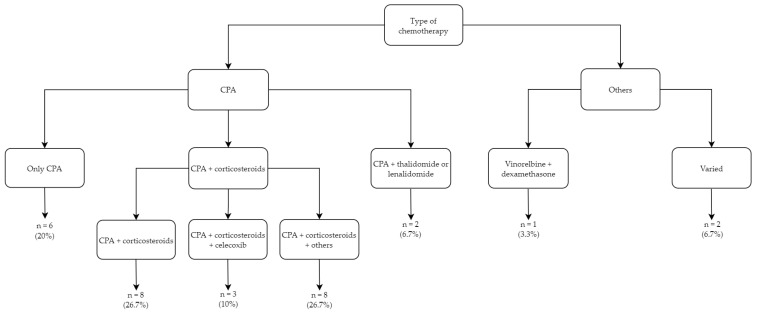
Details of the LDMC regimens. CPA = cyclophosphamide.

CPA was integrated into 27 of the 30 (90%) regimens (Figure 4) [19,21,22,23,24,25,27,28,29,30,31,32,33,34,35,37,38,39,40,41,42,43,44,46,47,48,49]. While six studies (20%) described the effects of CPA monotherapy [21,22,38,41,43,46], in the majority of reports, CPA was partnered with corticosteroids (19/30; 63.3%) [19,23,24,25,27,29,30,31,32,33,34,35,37,39,40,42,44,48,49]. Among the CPA/corticosteroid combination therapy studies, eight (27%) did not add further agents [23,24,27,31,35,37,40,49], the COX2 inhibitor celecoxib was added in three (10%) trials [19,34,44], and eight (27%) studies included other drugs (i.e., methotrexate, tegafur-uracil, etoposide, estramustine phosphate, and capecitabine) [25,29,30,32,33,39,42,48]. In two studies, CPA was combined with either thalidomide or lenalidomide (7%) [28,47]. 

Of the studies without a CPA backbone, a variety of agents were used. Di Desidero et al. studied vinorelbine with dexamethasone [26], Kubota et al. studied cisplatin and tegafur-uracil [36], and Tralongo et al. studied docetaxel or vinorelbine [45]. Of note, 24 of 30 (80%) treatment regimens comprised oral medications only [19,21,22,23,24,26,27,28,31,32,33,34,35,37,38,39,40,41,42,43,44,46,47,49]. 

### 3.4. Outcomes

**Figure 5:**
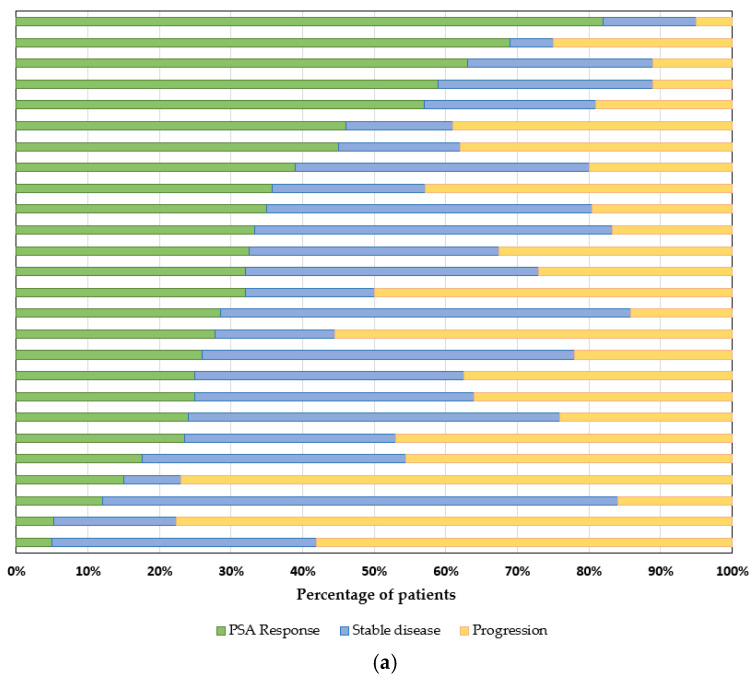

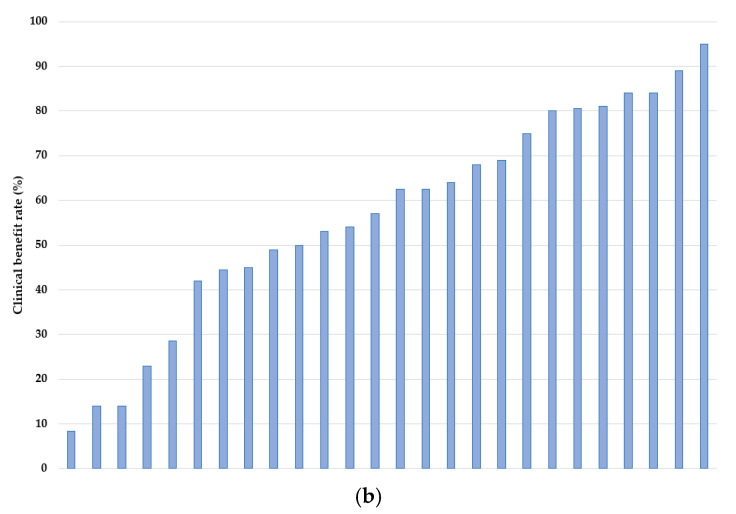
(**a**) Biochemical response assessment based on prostate specific antigen (PSA) across 26 informative studies. PSA response was defined as a ≥50% treatment-related PSA decrease compared with baseline. (**b**) Clinical benefit rate (%) across 26 informative studies.

To compare the effectiveness of the various LDMC regimens used, we extracted prostate-specific antigen (PSA) response rates and clinical benefit rates. Twenty-six trials provided information regarding patients’ PSA levels (Figure 5a) [19,21,22,24,25,26,27,28,29,30,31,32,33,34,36,37,38,39,40,41,42,44,45,46,47,49]. The mean ± SD PSA response rate (i.e., at least a 50% treatment-related PSA decrease compared with baseline) was 33 ± 19.1%, while another 32.2 ± 16.5% of patients achieved stable PSA readings. One third of study patients (33.5 ± 19.4%) did not experience any biochemical benefit from LDMC. 

The mean clinical benefit rate reported across 26 studies was 56.8 ± 24.5% (range from 8.3% to 95.0%) (Figure 5b). Of note, the publications used variable definitions of “clinical benefit”, with the most common being “sustained (≥6 months) absence of biochemical, clinical, and/or radiological progression”.

Twenty studies reported the median overall survival of patients on LDMC regimens [19,21,23,25,26,29,30,31,34,35,36,37,39,40,41,43,44,46,47,49]. Fontana et al. observed the shortest median survival of 3.3 (95%CI: 2.2–4.2) months [29], while Derosa et al. described the longest median survival of 33.3 (95%CI: 23–35.6) months [25]. The median of medians of reported overall survival was 16.2 months. 

### 3.5. Toxicities and Adverse Events

Twenty of the thirty studies used varying versions of the National Cancer Institute Common Toxicity Criteria for Adverse Events (NCI-CTCAE) to grade toxicities observed among study participants undergoing LDMC [19,21,22,23,25,26,27,29,30,32,34,36,37,38,39,40,42,43,44,46]. One of the studies used criteria set by the World Health Organization (WHO) [45], while four studies reported adverse events without indicating the type of criteria used [28,47,48,49]. Five studies did not include information regarding toxicities and adverse events in relation to LDMC [24,31,33,35,41]. 

**Figure 6:**
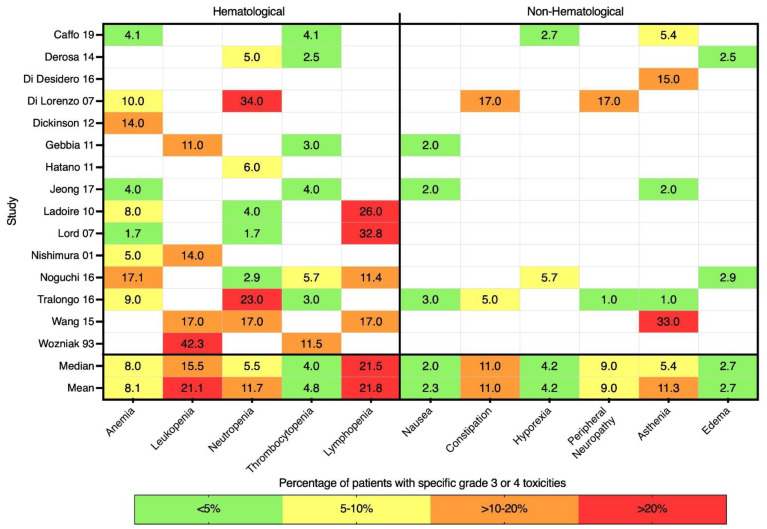
Heatmap of grade 3 and 4 toxicities observed in LDMC studies.

Figure 6 shows the percentage of study patients that experienced specific grade 3 or 4 (i.e., severe) toxicities reported in 15 informative clinical trials. Overall, hematological toxicities were more common than non-hematological adverse events. Instances of severe anemia were reported in nine trials, with a median of 8% of patients affected [21,27,28,34,37,38,42,43,45]. Grade 3/4 neutropenia was reported in eight studies, with a median of 5.5% of patients affected [25,27,32,37,38,43,45,47]. In the four studies listing severe lymphopenia, on average, around 20% of patients were affected. Asthenia was the most reported severe non-hematological adverse event, listed by five trials, with a median of 5.4% of patients affected [21,26,34,45,47].

**Figure 7:**
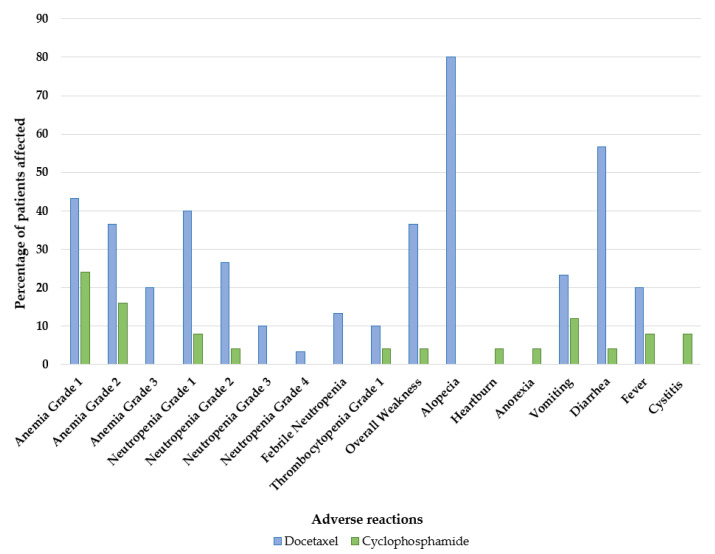
Depiction of the incidence of adverse events reported in patients undergoing conventional docetaxel versus metronomic cyclophosphamide therapy as reported by Vorob’ev et al., 2011 [46].

Vorob’ev et al.’s retrospective study compared the side effects of MTD docetaxel (75 mg/m^2^ administered intravenously every three weeks; *n* = 30 patients) versus LDMC CPA (50 mg by mouth daily; *n* = 25 patients) [46]. There were far fewer and less severe side effects reported in the CPA cohort (using NCI-CTCAE version 3) in comparison with patients treated with docetaxel (Figure 7). While a high percentage of patients treated with docetaxel were affected by diarrhea, alopecia, grade 1–3 anemia, and grade 1–4 neutropenia, patients in the CPA cohort were primarily affected by grade 1–2 anemia and grade 1–2 neutropenia, without severe (i.e., grade 3 or 4) cytopenia. Moreover, 16.7% of the docetaxel cohort stopped treatment due to adverse events, but no patient treated with CPA discontinued treatment because of side effects. Despite the distinct toxicity profiles, the mean overall survival was similar (>15 months) for both cohorts. However, MTD docetaxel resulted in a higher PSA response rate (46.7%) than LDMC CPA (12%). MTD docetaxel treatment was also slightly favored over LDMC CPA in terms of quality of life, measured with the Functional Assessment of Cancer Therapy-Prostate (FACT-P) questionnaire, and rate of pain response, based on a visual analogue scale, although these results were not statistically significant.

## 4. Discussion

The present literature review of 30 studies of LDMC for prostate cancer encompassing 973 patients illustrates several key findings. First, more than half of patients experience PSA responses or PSA stability and draw a clinical benefit from LDMC. Second, CPA is the most commonly used cytotoxic agent for metronomic purposes in prostate cancer (27/30 trials), as is the case in other cancer types [50]. The LDMC CPA studies characterize this classical alkylating agent as a convenient (oral mode of administration), well tolerated, and affordable (off-patent) treatment option that can be administered alone or in combination with other agents. Third, prostate cancer LDMC studies report low rates of severe (i.e., grade 3 or 4) adverse events. Hematological toxicity, notably lymphopenia, was more frequently observed than non-hematological adverse events. However, neither myelosuppression nor lymphopenia appear to be associated with an increased rate of infections. Furthermore, the low rate of typically mild LDMC-associated side effects compares favorably with the higher rate of adverse events seen with conventional MTD chemotherapy, including high-grade adverse events, as documented by Vorob’ev et al. [46]. 

Our study also reveals shortcomings and unmet needs regarding the clinical development of LDMC in prostate cancer. Foremost, there are no definite phase III clinical trials documenting the benefit of LDMC in prostate cancer, unlike in other malignancies such as breast, head and neck, and colorectal cancer [51,52,53,54,55,56,57,58]. Moreover, the majority of the LDMC prostate cancer studies were single-arm trials describing relatively small and often heterogeneous patient cohorts. Second, LDMC was almost exclusively studied in later stages of prostate cancer and often after multiple lines of prior therapies, whereas phase III trials in other tumor types suggest that LDMC might be particularly suitable for maintenance therapy in earlier tumor stages [52,55,57]. Third, there are no validated predictive markers of response to LDMC [59]. However, anecdotal evidence of responses to metronomic CPA in patients with DNA repair deficient mCRPC warrant further study [60].

In current times, with limited resources and growing expenses for the treatment of early as well as advanced prostate cancer, drug costs are becoming an increasingly important consideration when choosing treatments options [61,62,63]. With the incidence of cancer surging and the rising economic burden of cancer treatment worldwide, there is a need for affordable treatment options [2,64]. This is especially important for resource-limited countries, where mortality rates due to prostate cancer are rising, while decreasing in the more developed countries [65]. LDMC is an attractive treatment option in this respect. In Bocci et al.’s outcome analysis and cost comparison for conventional versus LDMC for metastatic breast cancer, LDMC was found to be more cost-effective due to several factors: LDMC (i) can be taken orally at home instead of administered during hospital visits, (ii) has lower incidences of adverse events, thereby decreasing related hospital and other healthcare visits, and (iii) is associated with lower administrative and health care provision costs due to a reduced need for medical attention [66]. 

Our systematic literature search did not identify studies comparing LDMC with ARSi therapy. Based on available evidence reported in the present analysis, it appears improbable that LDMC alone may provide a similar benefit to ARSi in advanced prostate cancer. Furthermore, ARSi are convenient oral albeit expensive medications that rarely result in severe side effects, even in patients who are older or frail [12,13,16,17]. However, LDMC might make ARSi therapy more affordable when integrated into intermittent ARSi regimens (e.g., LDMC maintenance therapy following ARSi induction). Aside from cost savings such LDMC use might also improve patient outcome by targeting ARSi-resistant prostate cancer cells [67,68]. Similarly, preliminary breast and ovarian cancer evidence suggests a possible role for combining LDMC using alkylating agents with poly (ADP-ribose) polymerase (PARP) inhibitors in patients with DNA-repair-deficient prostate cancer [69,70].

When extracting information from the 30 studies of LDMC for prostate cancer numerous limitations became apparent. Aside from the aforementioned lack of randomized controlled phase III trials, one third of studies were retrospective, and the majority of reports comprised relatively small single-arm studies. The definition of outcome measures such as clinical benefit rate varied across studies, rendering comparisons difficult. Not all reports contained information on adverse events. Finally, a wide variety of metronomic regimens were tested, although the use of CPA was a common denominator.

## 5. Conclusions

In conclusion, LDMC is a well-tolerated and cost-effective form of cancer therapy with documented anti-mCRPC effects. Because of mild toxicities and simple oral administration, LDMC can be regarded as an alternative treatment option especially for patients who are older or unfit and who are unable to tolerate conventional mCRPC therapies such as taxane chemotherapy. LDMC might also be considered in situations where ARSi are not available or affordable. Phase III trial evidence is needed to position LDMC with respect to other mCRPC therapies. 

## Figures and Tables

**Table 1:** Study characteristics.

First Author Name	Years	Study Type	Location	N	Age, Median (Range)	Reference
Caffo et al.	2019	retrospective	Italy	8	74 (56–95)	[21]
Calcagno et al.	2016	prospective	France	14	69 (57–82)	[22]
Calvani et al.	2019	retrospective	Italy	14	75 (56–87)	[23]
Dabkara et al.	2018	retrospective	India	16	74.5 (59–83)	[24]
Derosa et al.	2014	prospective	Italy	17	72 (52–79)	[25]
Di Desidero et al.	2016	prospective	Italy	17	73 (63–86)	[26]
Di Lorenzo et al.	2007	prospective	Italy	18	67 (46–75)	[27]
Dickinson et al.	2012	retrospective	UK	21	75 (N/A)	[28]
Fontana et al.	2009	prospective	Italy	23	74.5 (54–91)	[29]
Fontana et al.	2010	retrospective	Italy	24	83 (78–92)	[19]
Gebbia et al.	2011	prospective	Italy	25	72 (56–83)	[30]
Glode et al.	2003	retrospective	USA	25	72.6 (54–88)	[31]
Hatano et al.	2011	retrospective	Japan	25	71 (49–90)	[32]
Jellvert et al.	2011	prospective	Sweden	28	60 (45–75)	[33]
Jeong & Lee	2017	prospective	Korea	28	71 (49–88)	[34]
Knipper et al.	2019	retrospective	Germany	28	78 (N/A)	[35]
Kubota et al.	2017	prospective	Japan	29	74.2 (66–88)	[36]
Ladoire et al.	2010	prospective	France	32	74 (55–88)	[37]
Lord et al.	2007	prospective	UK	35	69 (51–86)	[38]
Meng et al.	2012	retrospective	China	38	72.8 (69–78)	[39]
Nelius et al.	2010	prospective	USA	39	68 (42–85)	[40]
Nicolini et al.	2004	prospective	Italy	41	72 (62–84)	[41]
Nishimura et al.	2001	prospective	Japan	43	70 (50–82)	[42]
Noguchi et al.	2016	prospective	Japan	49	68.6 (48–80)	[43]
Orlandi et al.	2013	retrospective	USA	52	81 (52–92)	[44]
Tralongo et al.	2016	prospective	Italy	57	77 (72–82)	[45]
Vorob’ev et al.	2011	retrospective	Russia	58	72.8 * (56–85)	[46]
Wang et al.	2015	prospective	USA	58	76 (50–86)	[47]
Wozniak et al.	1993	prospective	USA	74	67 (55–78)	[48]
Yashi et al.	2014	prospective	Japan	37	75 (67.8–79.3)	[49]

* = mean; N = sample size, N/A = not available.

## Data Availability

Not applicable.
